# Supplementary material for: Chromothripsis during telomere crisis is independent of NHEJ, and consistent with a replicative origin
Source: Genome Res. 2019 May;29(5):737–49. doi: 10.1101/gr.240705.118 (PMC6499312; doi:10.1101/gr.240705.118)
Supplement: Supplemental Material [file supp_gr.240705.118_Supplemental_file_1.zip › contigs/annotated_contigs/DB46/contig.2.DB46_length_375_mean_cov_3.6.docx]

**DB46_length_375_mean_cov_3.6**

```
CCTGAGCTAACCATGCACTCTTGTTTCCCTTTTGCCAGTTTTATCACATAGTGGATGTTCTGTAGCCAAATGAAAAACATTTTAACAAT
 >chr18:56533945-56534069 - E=3e-63
AAAACCCTTGCATACTTGACGTCCTTCACTGCAGA|CG|TCCATGTTAAAGAACACTGGAGCCAGGGTGTTTGGAACTCTCAGCGCAAC
                                      >chr18:56535029-56535278 + E=1e-138
CTGGTTCAGGAGCACACACGCTCTCTTTTTCTTCTGTTCTTTTCTCTCAGCGATTCCCTGCCCCCACCCACCTTGACTCATTTTAAGGA

AGGTCTAGGAAGGAGGTTGCCAACTTGTCAGTAGAATCTTCTGATGCCTTTGACTGTGAGTTAGAATTTTAGGGTTTGTGTTCTCCCAT

CTCCCATTTATTTCTTCTCTT
```
